# Sex differences in antipsychotic efficacy and side effects in psychosis

**DOI:** 10.1192/j.eurpsy.2023.122

**Published:** 2023-07-19

**Authors:** G. Erzin

**Affiliations:** Psychiatry, Ankara Dışkapı Training and Research Hospital, Ankara, Türkiye

## Abstract

**Abstract:**

There are a lot of studies investigating gender differences in the side effects of first and second-generation antipsychotic drugs. Even though some side effects are seen at higher rates in women, it is not determined yet the influence of gender on the efficacy and side effects of antipsychotic agents is still not well clear. In this review, we give a brief overview of antipsychotic side effects leading probably to distinguished clinical outcomes in both sexes.

**Disclosure of Interest:**

None Declared

